# Etymologia: *Haematospirillum jordaniae*

**DOI:** 10.3201/eid2904.220831

**Published:** 2023-04

**Authors:** Clyde Partin

**Affiliations:** Emory University, Atlanta, Georgia, USA

**Keywords:** etymologia, *Haematospirillum jordaniae*, bacteria, Delaware Public Health Laboratory, Jean G. Jordan, Orange Book, Special Bacteriology Reference Laboratory, Centers for Disease Control and Prevention, CDC

## *Haematospirillum jordaniae* [Hae.ma.to.spi.ril′lum jor.da′ni.ae]

**Figure 1:**
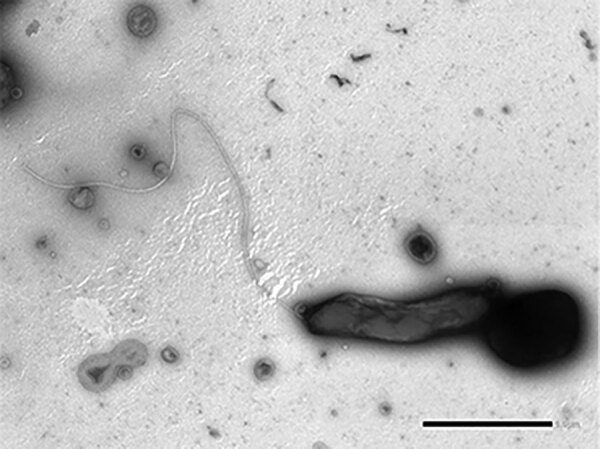
*Haematospirillum jordaniae* from a human blood sample. Scale bar indicates 1 μm. Source: (*5*).

For the sesquipedalian term Haematospirillum, Haema is derived from the Greek *haima*, meaning blood. Spirillum is derived from Medieval Latin in the mid-13th century Latin (*spiralis*), French in the 1550s (*spiral*), and Greek (*speira*). All suggest a winding or coil. A New Latin reference book entry in 1875 implied a little coil (Figure 1).

**Figure 2:**
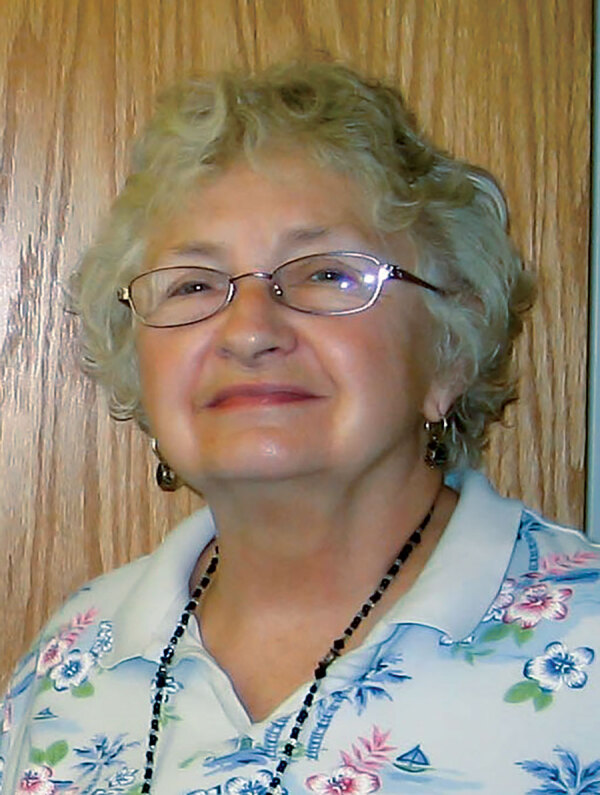
Jean G. Jordan. Photograph provided by the Special Bacteriology Reference Laboratory, Division of High-Consequence Pathogens and Pathology, National Center for Emerging and Zoonotic Infectious Diseases, Centers for Disease Control and Prevention.

Isolated from human blood, *Haematospirillum jordaniae* was reported as a novel genus and species in 2016 by Centers for Disease Control and Prevention (CDC) scientist Ben W. Humrighouse and his laboratory team, which included Jean G. Jordan, a microbiologist (Figure 2). This gram-negative bacterium was isolated 14 times in 10 states during 2003‒2012 before its identification in 2016.

*H. jordaniae* was previously considered an environmental bacterium with limited pathogenicity, but increasing numbers of isolates indicated a possible emerging pathogen. All cases occurred in male patients, and the pathogen showed a predilection for infecting lower leg injuries. In 2018, Hovan and Hollinger reported a case of infection in a Delaware man who, in 2016, had sepsis from a lower leg wound. The organism isolated was identified at the CDC Special Bacteriology Reference Laboratory (SBRL) in the Division of High-Consequence Pathogens and Pathology, National Center for Emerging and Zoonotic Infectious Diseases.

**Figure 3:**
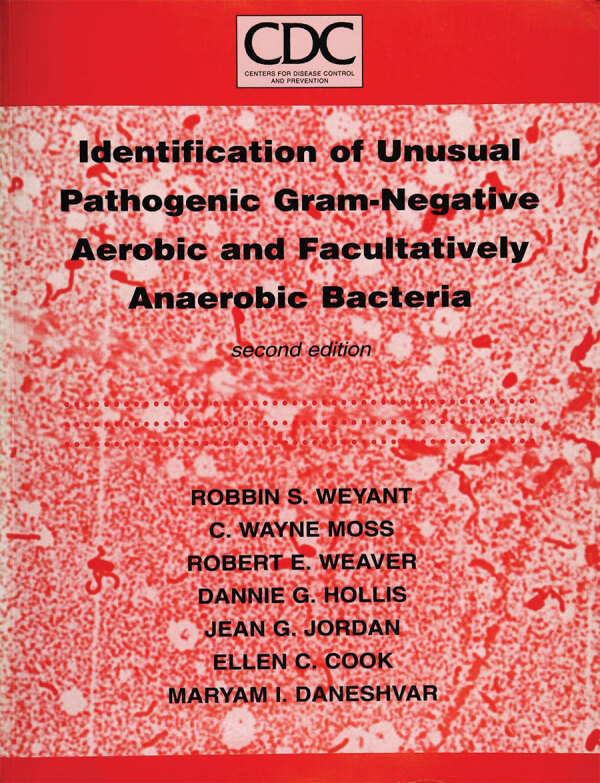
The Orange Book (*7*). Jean G. Jordan was one of the original authors.

Jordan, who helped identify an *H. jordaniae* sample in 2010, was honored by having the species named after her. She spent 52 years at CDC and was one of the authors of the “The Orange Book,” the standard reference for bacterial special pathogens, more formally known as “Identification of Unusual Pathogenic Gram-Negative Aerobic and Facultatively Anaerobic Bacteria (Figure 3).” Her colleagues fondly recall Jordan, noting, “Jean was an integral part of SBRL’s founding. Although not necessarily a well-known person, she was the behind the scenes expert who never wanted any special credit.”
